# An Artificial Neural Network–Based Pediatric Mortality Risk Score: Development and Performance Evaluation Using Data From a Large North American Registry

**DOI:** 10.2196/24079

**Published:** 2021-08-31

**Authors:** Niema Ghanad Poor, Nicholas C West, Rama Syamala Sreepada, Srinivas Murthy, Matthias Görges

**Affiliations:** 1 Research Institute BC Children’s Hospital Vancouver, BC Canada; 2 Department of Electrical Engineering and Computer Science Technische Hochschule Lübeck Lübeck Germany; 3 Department of Anesthesiology, Pharmacology & Therapeutics The University of British Columbia Vancouver, BC Canada; 4 Department of Pediatrics The University of British Columbia Vancouver, BC Canada

**Keywords:** artificial intelligence, risk assessment, decision support techniques, intensive care unit, pediatric, decision making, computer-assisted

## Abstract

**Background:**

In the pediatric intensive care unit (PICU), quantifying illness severity can be guided by risk models to enable timely identification and appropriate intervention. Logistic regression models, including the pediatric index of mortality 2 (PIM-2) and pediatric risk of mortality III (PRISM-III), produce a mortality risk score using data that are routinely available at PICU admission. Artificial neural networks (ANNs) outperform regression models in some medical fields.

**Objective:**

In light of this potential, we aim to examine ANN performance, compared to that of logistic regression, for mortality risk estimation in the PICU.

**Methods:**

The analyzed data set included patients from North American PICUs whose discharge diagnostic codes indicated evidence of infection and included the data used for the PIM-2 and PRISM-III calculations and their corresponding scores. We stratified the data set into training and test sets, with approximately equal mortality rates, in an effort to replicate real-world data. Data preprocessing included imputing missing data through simple substitution and normalizing data into binary variables using PRISM-III thresholds. A 2-layer ANN model was built to predict pediatric mortality, along with a simple logistic regression model for comparison. Both models used the same features required by PIM-2 and PRISM-III. Alternative ANN models using single-layer or unnormalized data were also evaluated. Model performance was compared using the area under the receiver operating characteristic curve (AUROC) and the area under the precision recall curve (AUPRC) and their empirical 95% CIs.

**Results:**

Data from 102,945 patients (including 4068 deaths) were included in the analysis. The highest performing ANN (AUROC 0.871, 95% CI 0.862-0.880; AUPRC 0.372, 95% CI 0.345-0.396) that used normalized data performed better than PIM-2 (AUROC 0.805, 95% CI 0.801-0.816; AUPRC 0.234, 95% CI 0.213-0.255) and PRISM-III (AUROC 0.844, 95% CI 0.841-0.855; AUPRC 0.348, 95% CI 0.322-0.367). The performance of this ANN was also significantly better than that of the logistic regression model (AUROC 0.862, 95% CI 0.852-0.872; AUPRC 0.329, 95% CI 0.304-0.351). The performance of the ANN that used unnormalized data (AUROC 0.865, 95% CI 0.856-0.874) was slightly inferior to our highest performing ANN; the single-layer ANN architecture performed poorly and was not investigated further.

**Conclusions:**

A simple ANN model performed slightly better than the benchmark PIM-2 and PRISM-III scores and a traditional logistic regression model trained on the same data set. The small performance gains achieved by this two-layer ANN model may not offer clinically significant improvement; however, further research with other or more sophisticated model designs and better imputation of missing data may be warranted.

## Introduction

### Background

The use of risk models in medicine enables timely and more targeted interventions for a given patient and facilitates benchmarking quality of care and conduct of clinical studies [1]. It is often necessary to quantify the severity of illness in the pediatric intensive care unit (PICU). Estimating the probability of mortality or expected length of stay from early admission data with such risk models is mainly used for quality improvement and benchmarking; however, it might enable a clinician to make objective medical decisions regarding the state of the patient, the necessary level of care, possible treatments, discharge plans, or expected costs [2-4].

PICUs are data-rich environments with a wide range of physiological variables that are responsive to interventions over short periods and outcomes that are well-defined and generally quantifiable [5]. Thus, the PICU provides fertile ground to develop and test prediction models of risks and outcomes. A score, which is quick and pragmatic to use, can enable the timely identification of adverse conditions and may be used to tailor appropriate interventions [6]. Two commonly encountered pediatric risk scores are the pediatric index of mortality 2 (PIM-2) [2] and pediatric risk of mortality III (PRISM-III) [1]. Both are derived from logistic regression models, which estimate mortality risk and have been validated with respective areas under the receiver operating characteristic curves (AUROCs) of 0.90 and 0.89 [1,7].

Increased computing capabilities, big data, and machine learning algorithms enable the application of artificial intelligence (AI) for clinical decision support [8]. Artificial neural networks (ANNs), a subtype of AI, can be used in different medical areas and have been shown to outperform physicians in diagnosis based on medical imaging or data from electronic medical records [9-12]. A recurrent neural network is a type of ANN that is most commonly used for sequential data. An ANN-based cardiac risk score, which used the recurrent neural network approach, was able to detect small changes in an electrocardiogram segment, which cannot be found by visual inspection [11]; another was used to classify clinical time series data for pediatric patients in critical care [12].

The clinical adoption of ANN-based risk models relies on gaining physicians’ trust in the use of AI [13,14], which may include, but is not limited to, demonstrating better performance than traditional regression approaches.

### Objectives

The primary aim of this study is to examine the performance of an ANN-based approach compared to that of traditional approaches based on logistic regression models when applied to estimating the risk of mortality in children admitted to PICU with suspected sepsis. We developed an ANN model using features required in the PIM-2 and PRISM-III models to predict mortality outcomes (died or survived) in a large North American registry data set and evaluated the ANN’s performance using the AUROC. We compared its performance with the benchmark PIM-2 and PRISM-III scores, as well as a logistic regression model, trained on the study data set, which used the same features as PIM-2 and PRISM-III.

## Methods

### Study Design and Approval

In this study, we used data from a North American PICU registry to compare the performance of an ANN model with PIM-2 and PRISM-III scores. The data set was obtained from Virtual Pediatric Systems (VPS), LLC, a registry of prospectively collected records from 130 PICUs in the United States and Canada. This is a secondary analysis of data obtained for a different purpose—to develop a simple risk stratification score for children with sepsis [6]. Ethical approval for the study was obtained from the University of British Columbia/Children’s and Women’s Health Centre of British Columbia Research Ethics Board (H15-01398). The requirement for written informed consent was waived by the research ethics board, as this study was a secondary analysis of registry data. This manuscript has been prepared in accordance with the guidelines for Transparent Reporting if a multivariable prediction risk model for Individual Prognosis or Diagnosis.

As sepsis diagnosis might not necessarily be known or documented *at the time of admission* to the PICU, we identified all children in the VPS data set whose diagnostic codes *at discharge* exhibited evidence of an infection, and combined with their admission to the PICU, this provides a reasonably strong indication for sepsis. This allowed us to create a representative data set of children with a high likelihood of sepsis.

### Study Data Set

#### Data Available for Analysis

The analyzed data set included data on PICU admissions between January 1, 2009, and December 31, 2014. Data were available from 102,945 children, of whom 4068 died (mortality rate 3.95%). Each entry included a variety of vital signs, laboratory tests, and other clinical information, including the variables required to calculate the PIM-2 and PRISM-III scores. The clinical data used in this analysis were solely from early admission to the PICU. Hence, the longer the length of stay, the less associated these predictors were with the outcome under investigation: mortality or survival at PICU discharge.

Although the variables for PIM-2 and PRISM-III were collected from the same source, these models captured data from different sampling windows. For any given PICU admission, the VPS data set provides a single measurement for each variable used by these 2 risk scores as required for their respective calculations.

#### PRISM-III Variables and Sampling Window

PRISM-III uses the highest or lowest values of systolic blood pressure, heart rate, temperature, mental status, pupillary reflexes, acidosis, pH, P_CO2_, total carbon dioxide (CO_2_), Pa_O2_, glucose, potassium, creatinine, blood urea nitrogen, white blood cell count, platelet count, and prothrombin time or partial thromboplastin time [1]. Values included were measured in the first 12 hours of PICU care; laboratory variables were also considered up to 2 hours before PICU admission.

#### PIM-2 Variables and Sampling Window

PIM-2 uses the first recorded values of systolic blood pressure, pupillary reaction to light, Pa_O2_, base excess, early mechanical ventilation (yes or no), elective PICU admission (yes or no), admission following surgery (yes or no), admission following cardiopulmonary bypass, high-risk diagnoses (nine options: cardiac arrest preceding intensive care unit (ICU) admission, severe combined immune deficiency, leukemia or lymphoma after first induction, spontaneous cerebral hemorrhage, cardiomyopathy or myocarditis, hypoplastic left heart syndrome, HIV infection, liver failure as the main reason for ICU admission, or neurodegenerative disorder), and low-risk diagnoses (five options: main reason for ICU admission of asthma, bronchiolitis, croup, obstructive sleep apnea, or diabetic keto-acidosis) [2]. Values included were measured in the first hour of PICU care starting at the time of the first face-to-face meeting of the patient with a PICU team member.

Not all vital signs were collected routinely for every patient, so the data set was only sparsely populated, and the vital signs used for calculating PIM-2 and PRISM-III scores were incomplete in some cases, for example, the Glasgow Coma Score (mental status) was missing from 60.2% (61,976/102,945) of cases. In the calculation of both PIM-2 and PRISM-III scores, missing vital signs are taken as a sign of being normal, that is, healthy, as such tests were not ordered or performed by the PICU team [1,2]. For example, a missing Glasgow Coma Score is interpreted as indicating a normal mental status and is input to the model as such. This assumption is discussed further in the *Limitations* section.

### Preprocessing

Preprocessing was performed in Python (v3.8.5; Python Software Foundation) to perform three tasks: (1) generate the training and test sets, (2) address missing values in the data set, and (3) generate new variables through data transformation.

#### Generation of Training and Test Sets

The total data set was initially divided into training and test sets using a stratified approach to ensure that the class ratio for mortality remained approximately equal for the training, test, and full data sets. ANN and logistic regression models were built on the training sets and evaluated on the test sets, and the results were compared against the PIM-2 and PRISM-III models. The overall data set was bootstrapped 100 times to generate the training and test sets.

#### Addressing Data Missingness Through Simple Substitution

The data set was examined for missing entries, and the missing values were imputed based on the feature type; specifically, the missing values in categorical features, such as pupillary reaction and coma status, were imputed using the most common value (mode). The missing values in numerical features, such as glucose or P_CO2_, were imputed using the median value, as most of these features did not follow a normal distribution. Median and mode approaches were used to build imputation models and fill the missing values in the training set, and these imputation models were applied to the test set separately to avoid a data leakage problem.

#### Generation of New Variables Through Data Transformation

We performed minimum-maximum normalization to normalize numerical data for the ANN and logistic regression models. The minimum and maximum values of each feature from the training set were used to normalize the data in the training and test sets to avoid data leakage. Dummy encoding was performed on categorical features that contained more than 2 distinct values, such as pupillary reaction, but all categorical features with only 2 distinct values were dichotomized to accommodate them in the machine learning models. We used thresholds defined by PRISM-III to define normal and abnormal values. PIM-2 does not have defined thresholds; however, it penalizes any diversion of a vital sign from its normal value continuously.

### Model Training

**Figure 1:**
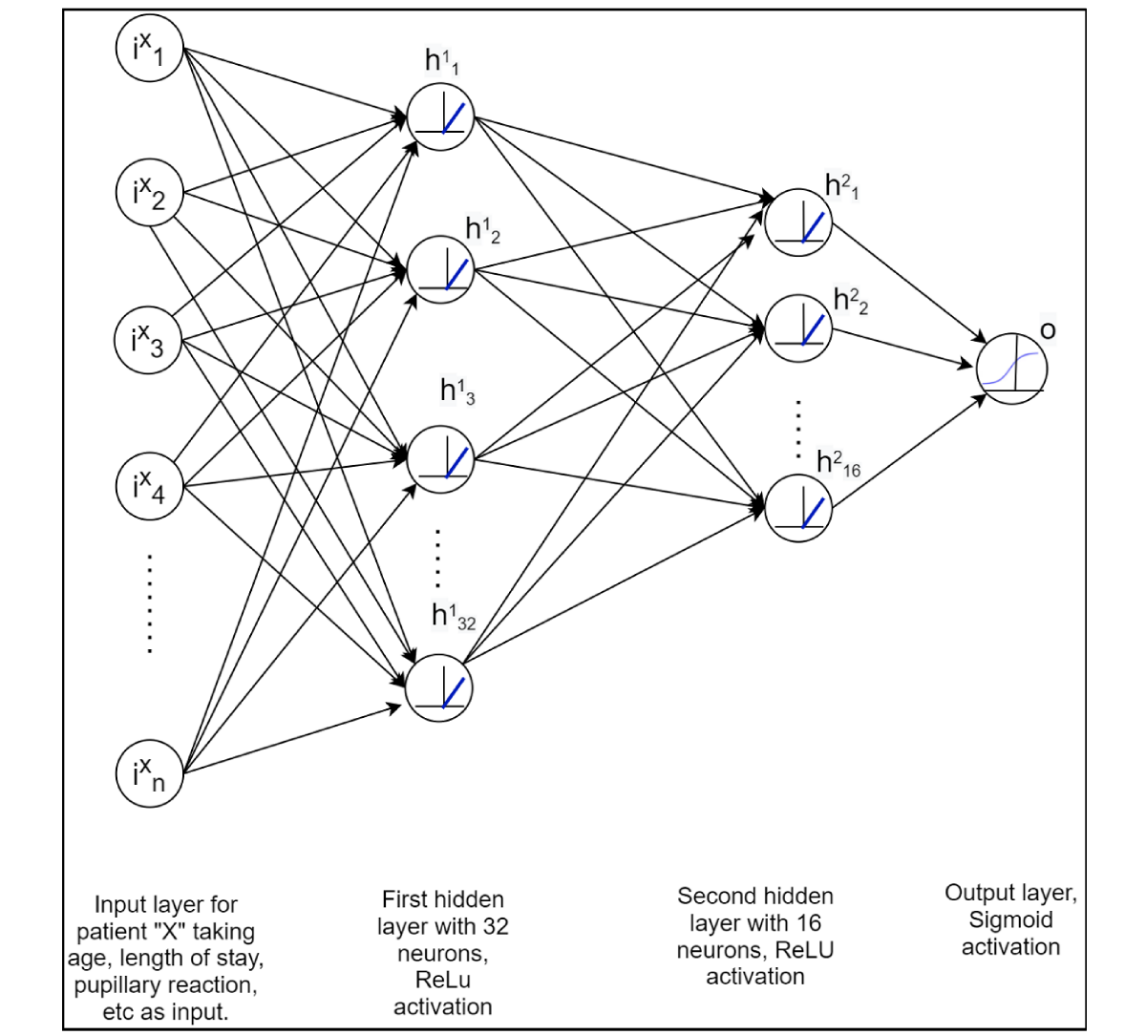
Artificial neural network architecture with two hidden layers: the node in input layer “iXy” processes data from pediatric intensive care unit admission “X” with feature “y” (such as age, length of stay, pupillary reaction, etc). The total number of features in the data set is denoted by “n.” The first and second hidden layers are represented by h1 and h2, respectively, with a subscript to denote the node number. The output layer has a single node (o), which shows probability of mortality for patient “X.” ReLU: rectifier linear unit.

We built an ANN model using the Keras framework on top of TensorFlow (Google Brain Team) in Python (Python Software Foundation), with training conducted in Jupyter notebook (IPython). The Python code files that were used to build the models and generate results are available in Multimedia Appendix 1. We used a grid approach to determine the optimum configuration while designing the neural network. We tested various configurations between 1 and 3 hidden layers and 8 and 32 neurons per hidden layer, with a rule-of-thumb approach to limit the number of hidden layer neurons to the neurons in the input layer. Through experimentation, we identified that a 2-layer ANN, with 32 nodes in the first hidden layer and 16 nodes in the second hidden layer, performed better than the other configurations we tested. Our final model consisted of 32 input features (consisting of the variables used in the PRISM-III [1] and PIM-2 [2] models; see the *Study Data Set* section), and the 2 hidden layers, with each node using rectifier linear unit activation functions; finally, a sigmoid activated dense layer was used to predict the mortality for each instance (Figure 1). The model was compiled using an *adam* optimizer with a binary cross-entropy loss function. While keeping the main network the same, we also evaluated the model with unnormalized data as well as a model with only a single hidden layer. We conducted training with a batch size of 32 and observed that the loss remained constant after 100 epochs.

The ANN model was trained with features used in the PIM-2 and PRISM-III models to predict the outcome (*died* or *survived*); AUROC was used as an evaluation metric while training the model. Finally, we developed a logistic regression model for comparison using the same features from PIM-2 and PRISM-III.

### Model Evaluation

The empirical range of AUROC scores was computed for each test set (obtained from bootstrap) using the *sklearn.metrics* function in Python. The test set that resulted in the median AUROC value was used to determine the optimum Youden index value. This threshold was then used to calculate the false positive rate (FPR) and false negative rate (FNR) for each test set, and the 95% empirical CIs were reported by pooling the results from all the test sets [15-17]; median and ranges of pooled results were reported for all other indices. We also reported the area under the precision recall curve (AUPRC) and its empirical 95% CI for each model. A Welch 2-sided *t* test was used to compare AUROC and AUPRC for model pairs.

To compare how the models performed at specific true positive rate (TPR) and FPR levels, we fixed the TPR values at 95%, 90%, and 85% and computed the corresponding median FPR values (from all the test sets) for ANN, logistic regression, PIM-2, and PRISM-III. Similarly, we also reported the median TPR results by fixing the FPR at 5%, 10%, and 15%.

## Results

### Data Set Characteristics

The data set included 102,945 children with infection admitted between 2009 and 2014, of whom 4068 died (3.95% mortality rate). The training sets contained 72,061 children, of whom a median of 2852 (range 2790-2903) died, equivalent to a 3.96% mortality rate; the test sets contained 30,884 children, of whom a median of 1216 (range 1165-1278) died, equivalent to a 3.94% mortality rate (Table 1).

**Table 1:** Overview of study population with demographics and risk factors split by outcome (N=102,945)^a^.

Characteristic			All (n=102,945)		Died (n=4068)		Survived (n=98,877)		Training					Testing		
									Died (n=2852)		Survived (n=69,209)		Died (n=1216)			Survived (n=29,668)
Males, n (%)			58,058 (56.39)		2186 (53.73)		55,872 (56.5)		1531 (53.68)		39,075 (56.46)		655 (53.87)			16,797 (56.62)
**Age**																
	Age (months), median (IQR)	28.9 (7.5-100.3)		39.3 (7.0-137.7)		28.6 (7.5-98.7)		37.8 (6.9-138.4)		28.4 (7.3-98.4)		42.45 (7.2-135.25)			29.3 (7.9-99.4)	
	<1 month, n (%)	4733 (4.6)		342 (8.41)		4391 (4.44)		245 (8.59)		3108 (4.49)		97 (7.98)			1283 (4.32)	
	1-23 months, n (%)	42,935 (41.71)		1400 (34.41)		41,535 (42.01)		980 (34.36)		29,195 (42.18)		420 (34.54)			12,340 (41.59)	
	2-5 years, n (%)	22,264 (21.63)		719 (17.67)		21,545 (21.79)		510 (17.88)		14,967 (21.63)		209 (17.19)			6578 (22.17)	
	6-12 years, n (%)	18,652 (18.12)		776 (19.07)		17,876 (18.08)		542 (19)		12,474 (18.02)		234 (19.24)			5402 (18.21)	
	13-18 years, n (%)	14,353 (13.94)		829 (20.39)		13,524 (13.68)		574 (20.13)		9461 (13.67)		255 (20.97)			4063 (13.69)	
	>18 years, n (%)	8 (0.01)		2 (0.05)		6 (0.01)		1 (0.04)		4 (0.01)		1 (0.08)			2 (0.01)	
**Primary diagnosis category, n (%)**																
	Respiratory	63,928 (62.1)		1404 (34.51)		62,524 (63.23)		968 (33.94)		43,696 (63.14)		436 (35.86)			18,828 (63.46)	
	Infectious	12,288 (11.94)		1387 (34.1)		10,901 (11.02)		979 (34.33)		7588 (10.96)		408 (33.56)			3313 (11.17)	
	Neurological	3589 (3.49)		162 (3.98)		3427 (3.47)		120 (4.21)		2447 (3.54)		42 (3.45)			980 (3.3)	
	Gastrointestinal	2248 (2.18)		103 (2.53)		2145 (2.17)		79 (2.77)		1518 (2.19)		24 (1.97)			627 (2.11)	
	Dermatologic	1769 (1.72)		30 (0.74)		1739 (1.76)		18 (0.63)		1219 (1.76)		12 (0.99)			520 (1.75)	
**Location before PICU^b^ admission, n (%)**																
	Inpatient	30,691 (29.81)		1752 (43.07)		28,939 (29.27)		1238 (43.41)		20,280 (29.3)		514 (42.27)			8659 (29.19)	
	Postoperative admission	18,435 (17.91)		576 (14.16)		17,859 (18.06)		412 (14.45)		12,447 (17.98)		164 (13.49)			5412 (18.24)	
**Resuscitation procedures**																
	Cardiac massage before PICU, n (%)	1863 (1.81)		588 (14.45)		1275 (1.29)		419 (14.69)		864 (1.25)		169 (13.9)			411 (1.39)	
	Mechanical ventilation within 24 hours, n (%)	53,903 (52.36)		3417 (84)		50,486 (51.06)		2405 (84.33)		35,258 (50.94)		1012 (83.22)			15,228 (51.33)	
	Mechanical ventilation within 1 hour, n (%)	42,940 (41.71)		2658 (65.34)		40,282 (40.74)		1863 (65.32)		28,161 (40.69)		795 (65.38)			12,121 (40.86)	
	Length of stay (days), median (IQR)	3.5 (1.7-8.0)		7.2 (2.2-21.4)		3.4 (1.7-7.8)		7.1 (2.2-21.0)		3.4 (1.7-7.8)		7.5 (2.4-22.7)			3.4 (1.6-7.8)	
	PRISM-III^c^ probability of death (%), median (IQR)	0.63 (0.3-1.6)		10 (1.7-44.6)		0.5 (0.3-1.4)		10.2 (1.7-47.8)		0.5 (0.3-1.4)		8.3 (1.6-39.2)			0.5 (0.3-1.4)	
	PIM-2^d^ probability of death (%), median (IQR)	1 (0.4-3.5)		5.2 (2.8-18)		1 (0.3-3.3)		5.3 (2.9-18.2)		0.9 (0.3-3.3)		4.8 (2.1-17.6)			0.9 (0.3-3.3)	
	Died, n (%)	4068 (3.95)		4068 (100)		0 (0)		2852 (100)		0 (0)		1216 (100)			0 (0)	

^a^Data are reported separately for the complete population and the training and test cohorts. Note that only the top 5 primary diagnosis categories are reported. In addition, note that only the initial 3 columns are true results; the remaining 4 are median values over the 100 data sets created.

^b^PICU: pediatric intensive care unit.

^c^PRISM-III: pediatric risk of mortality III.

^d^PIM-2: pediatric index of mortality 2.

As is commonly encountered in large clinical registries using clinical availability of routinely collected data, between 0.41% (424/102,945) and 80.27% (82,636/102,945) of entries were missing per feature required for PRISM-III: more commonly measured vital signs, such as systolic blood pressure and heart rate, had fewer missing values (424/102,945, 0.41%-517/102,945, 0.5%), whereas others were missing many entries, such as CO_2_ (49,837/102,945, 48.41% missing) and partial thromboplastin time (82,636/102,945, 80.27% missing). For PIM-2, only 2.05% (2115/102,945) of entries were missing the numerical feature systolic blood pressure, whereas base excess was missing in 84.73% (87,230/102,945) of entries, and both fraction of inspired oxygen and Pa_O2_ were missing from 94.18% (96,958/102,945) of the entries. On the other hand, there was no missing information in any of the binary features, such as high- or low-risk diagnosis and recovery from surgery, which are features required for the PIM-2 calculation.

### Model Performance: ANN Trained Using Imputed and Normalized Data

With the ANN trained on normalized data, the median FPR was mostly close to 18.4% (range 12.5%-30.8%) and the median FNR value was 24% (range 12.7%-33.2%; Table 2), with a median accuracy of 81.3% (range 69.9%-86.7%) on the test set.

**Table 2:** Performance characteristics of 4 different mortality prediction models^a^.

Prediction model	Threshold trigger (%)	False positive detections (FPR^b^), n (%)	Missed cases (FNR^c^), n (%)
PIM-2^d^	3.36	7278 (24.5)	377 (31.5)
PRISM-III^e^	2.21	1052 (3.5)^f^	651 (54.3)
Logistic regression	0.50	5142 (17.3)	317 (26.8)
ANN^g^	0.04	5467 (18.4)	289 (24.0)^f^

^a^Comparison of the pediatric index of mortality 2, pediatric risk of mortality III, a traditional logistic regression model, and artificial neural network–based approach. For each model, the threshold was selected by optimizing the Youden index.

^b^FPR: false positive rate.

^c^FNR: false negative rate.

^d^PIM-2: pediatric index of mortality 2.

^e^PRISM-III: pediatric risk of mortality III.

^f^The best value in this category.

^g^ANN: artificial neural network.

**Figure 2:**
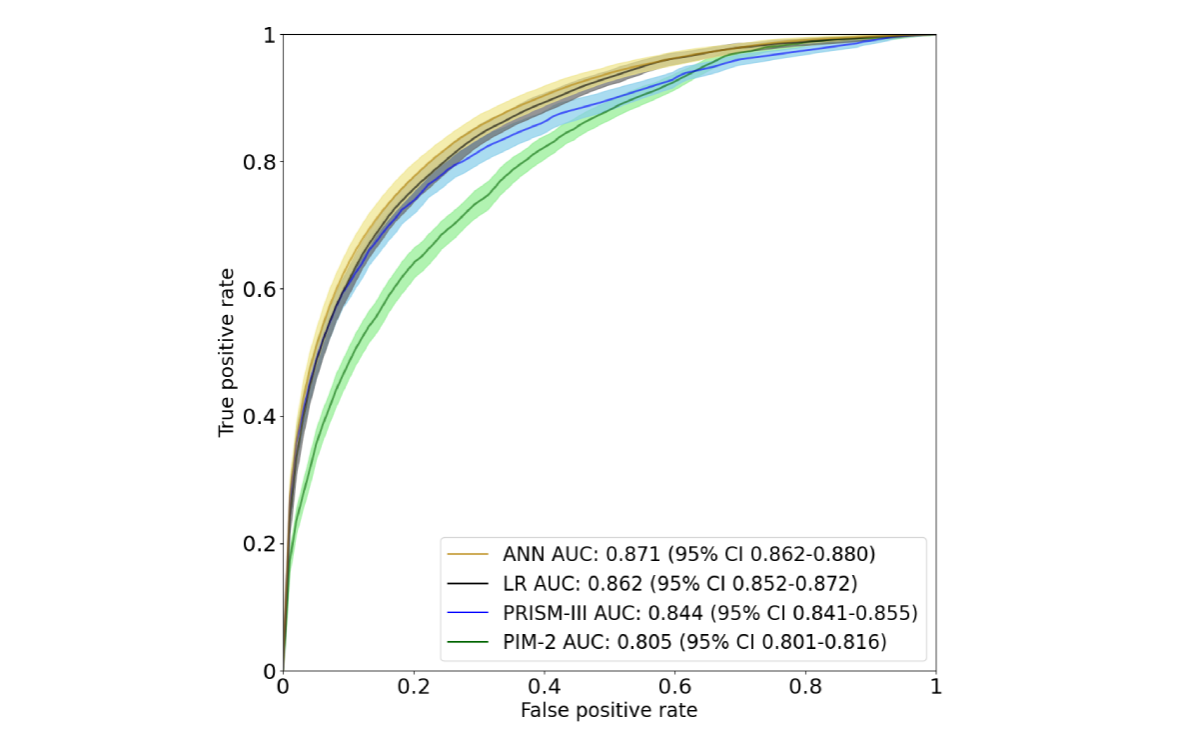
Receiver operating characteristic curves for four different mortality prediction models: pediatric index of mortality 2, pediatric risk of mortality III, logistic regression, and our best artificial neural network–based approach. The areas under the receiver operating characteristics curve and their 95% CI are indicated in the bottom-right corner. ANN: artificial neural network; AUC: area under the receiver operating characteristic curve; LR: logistic regression; PIM-2: pediatric index of mortality 2; PRISM-III: pediatric risk of mortality III.

The AUROCs for PIM-2 and PRISM-III were 0.805 (95% CI 0.801-0.816) and 0.844 (95% CI 0.841-0.855), respectively. The ANN (AUROC 0.871, 95% CI 0.862-0.880) performed better than both PIM-2 (*P*<.001) and PRISM-III (*P*<.001; Figure 2).

**Figure 3:**
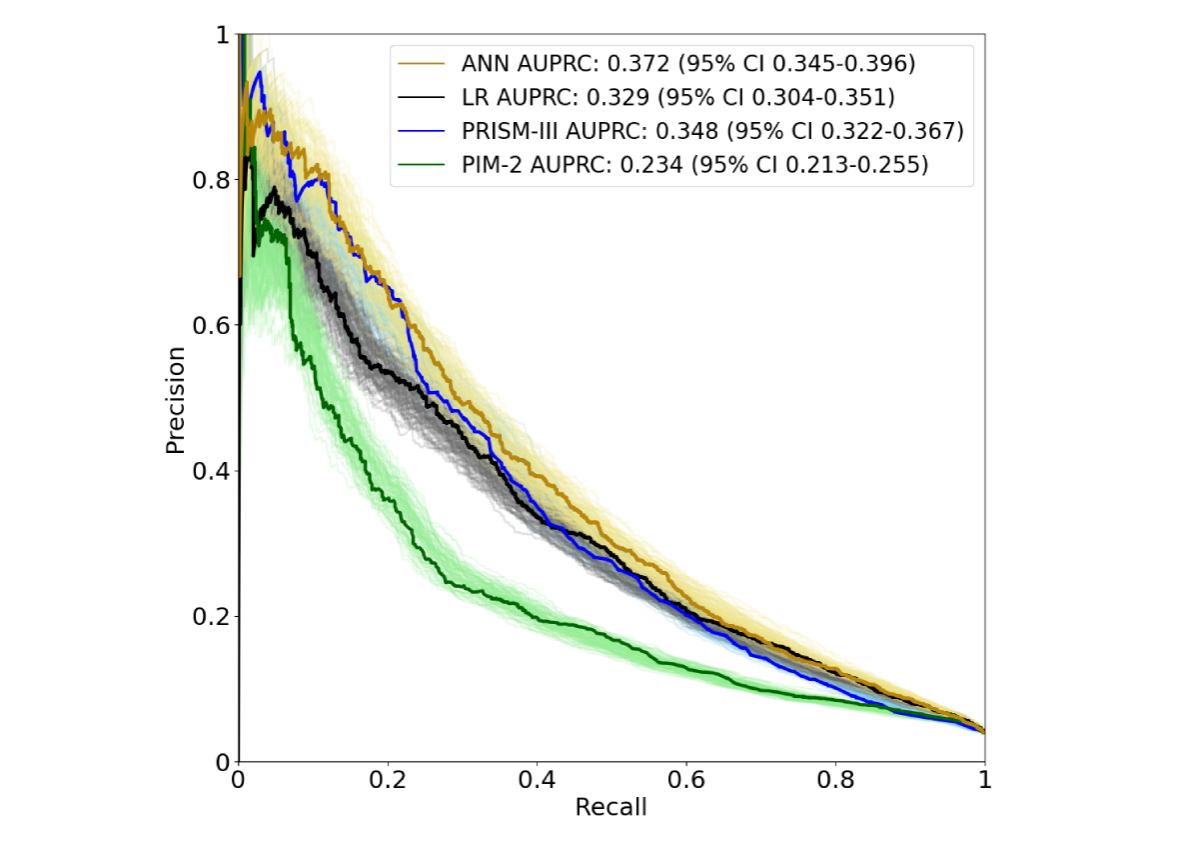
Precision recall curves for four different mortality prediction models: pediatric index of mortality 2, pediatric risk of mortality III, logistic regression, and our best artificial neural network–based approach. The areas under the precision recall curves and their 95% CI, are indicated in the top right corner. ANN: artificial neural network; AUPRC: area under the precision recall curve; LR: logistic regression; PIM-2: pediatric index of mortality 2; PRISM-III: pediatric risk of mortality III.

Similar results were observed using AUPRC, which indicated that the ANN (AUPRC 0.372, 95% CI 0.345-0.396) performed better than PIM-2 (AUPRC 0.234, 95% CI 0.213-0.255; *P*<.001) and PRISM-III (AUPRC 0.348, 95% CI 0.322-0.367; *P*<.001; Figure 3). The ANN achieved the highest TPR compared with the logistic regression, PIM-2, and PRISM-III when FPR was fixed at 5%, 10%, or 15%. Similarly, FPR was lowest for the ANN when TPR was fixed at 85% or 90% (Table 3). However, the logistic regression model showed the smallest FPR when TPR was fixed at 95%.

**Table 3:** Median true positive rate and median false positive rate of 4 different mortality prediction models^a^.

Prediction model	TPR^b^ (%)				FPR^c^ (%)		
	FPR fixed at 5%	FPR fixed at 10%	FPR fixed at 15%	TPR fixed at 95%		TPR fixed at 90%	TPR fixed at 85%
PIM-2^d^	35.6	48.1	56.8	64.6		54	44.2
PRISM-III^e^	48.8	60.5	68.4	66.8		49.4	36.7
Logistic regression	48.4	61.2	69.8	55.1^f^		41.6	31.4
ANN^g^	49.7^f^	62.6^f^	70.8^f^	56		41.1^f^	30.7^f^

^a^Comparison of the pediatric index of mortality 2, pediatric risk of mortality III, a traditional logistic regression model, and artificial neural network–based approach.

^b^TPR: true positive rate.

^c^FPR: false positive rate

^d^PIM-2: pediatric index of mortality 2.

^e^PRISM-III: pediatric risk of mortality III.

^f^The best value in this category.

^g^ANN: artificial neural network.

The lowest FPR observed at the Youden-optimized threshold point for any of the models evaluated was 3.5% using PRISM-III, with a corresponding FNR of 54.3% (Table 2). If we target an FPR of 3.5%, the corresponding FNRs for the other models were 68.7% for PIM-2, 55.8% for the logistic regression, and 54% for the ANN.

### Model Performance: Logistic Regression Using Imputed and Nonnormalized Data

The accuracy of the logistic regression model was 81.9% (range 81.4%-82.5%), with an FPR of 17.3% (range 17%-18.3%) and an FNR of 26.8% (range 23.7%-29.9%; Table 2). The AUROC was 0.862 (95% CI 0.852-0.872) and the AUPRC was 0.329 (95% CI 0.304-0.351). The logistic regression model also showed better performance, as measured by AUROC, than both PIM-2 (*P*<.001) and PRISM-III (*P*<.001; Figure 2), but PRISM-III performed better than the logistic regression model when evaluated using AUPRC (Figure 3).

Although the AUROCs of the ANN and logistic regression overlap, it was found that ANN performed better than logistic regression (*P*<.001).

### Model Performance: ANN Trained Using Imputed and Nonnormalized Data

The accuracy of the ANN model trained using the nonnormalized data set with imputed data was 82.5% (range 69.6%-89.2%). The FPR value was 17.9% (range 9.7%-31.1%), and the FNR value was 26.7% (range 13.9%-39.3%; Table 2). The AUROC was 0.865 (95% CI 0.856-0.874), which was lower than that of the model with normalized data (*P*<.001). The AUPRC value was 0.355 (95% CI 0.328-0.376).

Using nonnormalized data, the ANN model had an FPR of 16.8% (95% CI 14.8%-18.2%) at a TPR of 73.3% and achieved its highest TPR of 73.4% (95% CI 71.4%-75.6%) for an FPR of 17.3%.

## Discussion

### Principal Findings

#### Summary of Results

We created an ANN-based pediatric risk prediction score using the features included in PIM-2 and PRISM-III scores, which we trained on patients from a large North American multicenter pediatric cohort with presumed sepsis as identified by a discharge diagnosis of infection. The overall performance of the ANN model with binary cross-entropy loss was better than the PIM-2 and PRISM-III scores, with median AUROCs of 0.871 (ANN) versus 0.805 (PIM-2; *P*<.001) and 0.844 (PRISM-III; *P*<.001). It also performed better than a traditional logistic regression model that used the same features required by PIM-2 and PRISM-III. However, these performance gains may not represent a clinically significant improvement. Our evaluation of the ANN approach with a single hidden layer and nonnormalized data returned poorer results than the other models evaluated.

#### Improved Performance, but Is It Relevant?

Our highest performing ANN was significantly better, statistically, than PIM-2 and PRISM-III using the AUROC and AUPRC measures of performance. The ANN missed fewer cases than PIM-2, PRISM-III, and the logistic regression model (ie, the ANN had a lower FNR; Table 2) at their respective ideal thresholds, as determined by optimizing their respective Youden indices; however, its rate of false positive detections was higher than that of PRISM-III and marginally higher than that of the logistic regression model (ie, the ANN had a higher FPR) at these Youden-optimized thresholds. This may suggest an opportunity for further optimization and evaluation, but it should be noted that the ANN did not miss more cases than PRISM-III (ie, the ANN had an equivalent FNR) when the FPR was fixed at the value of 3.5% (PRISM-III’s Youden-optimized threshold). A direct comparison between models is challenging given that model selection will depend to a large extent on the clinical context; in some settings, a single objective (eg, to minimize FPR) may be the overriding concern, whereas in other cases, a balance of multiple objectives may be required (eg, to minimize both FPR and FNR).

Despite limited performance gains and increased robustness, the improvement may not be clinically relevant and is unlikely to overcome the initial concerns that physicians might have about the new model. The limited performance gains were not surprising. Although studies have proposed that ANNs outperform logistic regression models [12,18] or offer at least partially better performance [19], a recent systematic review of 71 studies found no superior performance of ANN over logistic regression models [20]. However, ANN-based models allow for the tuning of performance characteristics, which offers a potential advantage.

#### Trust Issues as a Barrier to ANN Use in Risk Modeling

The successful acceptance of AI-based risk models requires physicians’ willingness to accept AI models and the interpretability of those models. Although clinically improved performance might help this case, trust is a key element in acceptance, which is built (or lost) in a dynamic and evolving process [13,21]. Our failure to demonstrate a significant improvement in clinical performance will not help overcome the barriers to adoption.

Future AI-based risk models may need to become more interpretable to find acceptance [14], and the higher the risk, the more interpretability is needed to earn the trust. Including clinicians and patients in the development of AI models may be a step toward promoting acceptability and interpretability. Certification and licensure for AI models might also help build trust in model-based risk scores [22,23]. Finally, it may be useful to assure the user that the model is a tool and not a replacement for the clinician [13].

#### Challenges With Skewed Data

The working data set was skewed: only 3.95% (4068/102,945) of instances had the outcome as *died*. Local minima are a problem frequently associated with imbalanced data sets, and customized learning algorithms, cost functions, or external approaches (ie, resampling the data set) can be used to help overcome this problem [24]. Some ANNs tended to predict (mostly) everyone as a survivor; given the overall mortality rate of the population (4068/102,945, 3.95%), even assuming every patient will survive results in an accuracy of approximately 96%, but with an FPR of zero and an FNR of one. A traditional experimental setup with accuracy as an evaluation metric fails when building models with skewed data, as the models tend to be biased toward the majority class (here *survived*) [25]. This challenge can be addressed by modifying the cost function to maximize the AUROC of the model [25].

### Limitations

The main limitation of this work is the fact that out of several ANN-based models evaluated, only 1 type learned to discriminate between survival and death of patients effectively. Despite attempts to address the root cause (imbalance of outcomes in the data set), this suggests that the approaches selected may not have been optimal and that further network types and designs should be considered in future approaches. Following the initial positive outcomes with this model, secondary training on a data set can be used to fine-tune the ANN model.

The information included in the new models was limited to risk factors from PIM-2 and PRISM-III. By creating new features such as vital sign combinations or ratios [26], which in principle can be emulated by adding hidden layers, one might be able to provide another significant performance boost to the model. However, this did not seem to be the case in a recent sepsis prediction competition [27], where novel methods or applications seemed to be more promising than the creation of new features.

Another limitation was the relatively low number of complete patient entries in the VPS data set. Given that VPS is a curated data set, the potential reasons for this likely stem from local practices, such as tests not being required for clinical management in particular cases or it being generally decided that recording the results of these tests is optional​. Although it makes the creation and use of some modeling techniques more difficult, this is an unavoidable feature of real-world clinical data. Characterizing the missingness to inform modeling might offer a valuable approach, but such features may not be generalizable because they represent local patterns of practice. To use models without the filtering layer, simple imputation approaches were used; however, data were likely not missing at random, which invalidates some of the (median or mode imputation) approaches used. More sophisticated approaches for handling data missingness, such as multivariate imputation by chained equations, may yield better performance [28,29], as the substituted values are likely closer to specific cases than the overall population. Importantly, physicians should inform the treatment of missing values, which might boost confidence in the methods used. It might be possible to use a complete time series in an ANN instead of extreme values observed in a certain window, which could improve performance.

This study explored only a limited range of ANN design techniques. For example, we used rectifier linear unit activation in the hidden layers but did not evaluate the effect of other activation functions on model performance; similarly, we used the *adam* optimizer to identify the optimal ANN architecture but did not evaluate alternative optimizers. Thus, more exhaustive experimentation may yield improved performance results. Similarly, Youden index was used as a pragmatic approach to identify the optimal cut off by maximizing the models’ true positive and true negative rates. However, selecting the appropriate operating point for clinical implementation should consider alternative approaches to finding the optimal threshold and would also require a more nuanced evaluation of clinical priorities, which might, for example, penalize missed cases over false positives.

A major limitation to the development of a new risk score is the lack of recognized clinically acceptable performance criteria to assess the utility of integrating ANN-based risk scores into daily clinical routines. In their absence, it is difficult to make a clear statement on the clinical utility of models with slightly better performance compared with existing risk scores.

### Conclusions

This study examined the performance of ANN models over logistic regression-based models to estimate the risk of mortality in the PICU. A simple 2-layer ANN demonstrated better performance than traditional logistic regression, PIM-2, and PRISM-III; the statistically significant improvement in performance may not be clinically significant. Further work, including involvement of physicians in defining performance thresholds, better handling of data missingness, and possibly the use of more sophisticated ANN-modeling methods, will be required to achieve meaningful advances to guide decision-making in the care of critically ill children.

## Appendix

Multimedia Appendix 1 Zipped Python code files were used to build the models and generate the results.

## Abbreviations

AI: artificial intelligence
ANN: artificial neural network
AUPRC: area under the precision recall curve
AUROC: area under the receiver operating characteristic curve
FNR: false negative rate
FPR: false positive rate
ICU: intensive care unit
PICU: pediatric intensive care unit
PIM-2: pediatric index of mortality 2
PRISM-III: pediatric risk of mortality III
TPR: true positive rate
TRIPOD: Transparent Reporting if a multivariable prediction risk model for Individual Prognosis or Diagnosis
VPS: Virtual Pediatric Systems
